# Tuberculosis in Dairy Cattle, and How Shall We Get Rid of It?1Read before the Annual Meeting of the Pennsylvania State Veterinary Medical Association, March, 1899.

**Published:** 1899-07

**Authors:** N. E. Reinhart

**Affiliations:** Pottstown, Pa.


### TUBERCULOSIS IN DAIRY CATTLE, AND HOW SHALL WE GET RID OF IT?[1]

By N. E. REINHART, V.S.,

POTTSTOWN, PA.

TUBERCULOSIS—identical with the disease known as consumption in the human family. The sameness between bovine tuberculosis and the same disease in man, combined with the mortality of human beings from this disease, has raised the question in all civilized countries as to how far animal tuberculosis was responsible. If this disease is transmissible from animals to man, how does this transmission take place, as not many persons come in direct contact with cattle? It must be either through meat or milk, or both, and products manufactured from them. A great variety of opinions on this subject are recorded. Some claim that the meat and milk from cows in which the muscles and bones show no deposit, or the udder no tuberculosis, are not dangerous diseases; others that the bones are seldom affected, and so long as there are no tuber-

[1] Read before the Annual Meeting of the Pennsylvania State Veterinary Medical Association, March, 1899.

cular deposits in either the bones or muscles the flesh of such animals is, in their opinion, not dangerous and is fit for food. But experiments have shown that tubercle bacilli have been found in milk taken from udders in which no sign of disease existed. Do not tubercle bacilli circulate in the blood and lymph? Can they be detected in the muscles? Whether tubercle bacilli are found in muscle juices independent of any deposits I am not prepared to say, but we do find tubercular deposits in muscles of the shoulders and flanks. Some writers say the deposits are rarely found in the bones. I have frequently found the ends of long bones of the limbs affected, and lameness caused by it.

There is great difference of opinion how far an animal must be affected to render it and its products unfit for use, but I do not think we should let the value of an animal or many animals be taken into consideration. If diseased at all they should be condemned and killed.

The work of the State Livestock Sanitary Board has done much to better the condition of our dairy herds, by ordering tests with tuberculin, as that is the only way of detecting many cases which show no sign of disease until it is used and the diagnosis confirmed by the autopsy. When the law went into effect under which the State Sanitary Board is working, I, like many others, did not think much tuberculosis existed among our cattle. Although practising veterinary medicine, I did not see much of it, as veterinarians were not often consulted in these cases. The farmer saw his cow failing, and sold her to a dealer in low-priced cattle, she going for bologna. I have seen many dairies tested in which no diseased animals were found, and others in which many were condemned. I think all cattle should be tested with tuberculin, and in this way we would rid our herds of this disease.

